# Pharmacological Treatments Available for Immune-Checkpoint-Inhibitor-Induced Colitis

**DOI:** 10.3390/biomedicines10061334

**Published:** 2022-06-06

**Authors:** Sae Ohwada, Keisuke Ishigami, Noriyuki Akutsu, Hiroshi Nakase

**Affiliations:** Department of Gastroenterology and Hepatology, Sapporo Medical University School of Medicine, Hokkaido 060-8556, Japan; saeoowada@gmail.com (S.O.); keisuke.ishigami@gmail.com (K.I.); akutsu@sapmed.ac.jp (N.A.)

**Keywords:** immune checkpoint inhibitor, immune-related adverse events, irAE colitis, corticosteroid, infliximab, vedolizumab, biologics

## Abstract

Immune checkpoint inhibitor treatment has shown revolutionary therapeutic effects in various carcinomas. However, immune-related adverse events (irAE) following this treatment can sometimes lead to treatment discontinuation. One such frequently encountered adverse event is immune-related colitis (irAE colitis). Corticosteroids (CS) are the first-line treatment for irAE colitis, but we often encounter CS-refractory or -resistant cases. The application of multiple biologics has been proposed as a therapy to be administered after CS treatment; however, the efficacy and safety of biologics for patients with irAE colitis who do not respond to CS have not been established. This review summarizes the treatment regimens available for irAE colitis, focusing on the mechanism of action of corticosteroids, infliximab, vedolizumab, and other drugs.

## 1. Introduction

The application of immune checkpoint inhibitors (ICIs), targeting the programmed cell death 1 (PD-1)/programmed cell death ligand 1 (PD-L1) and cytotoxic T-lymphocyte antigen 4 (CTLA-4) pathways, holds immense promise for cancer treatment. ICIs exert anti-tumor activity by reactivating host-specific cytotoxic T cells that are suppressed by the PD-1/PD-L1 and CTLA-4 pathways. ICIs have contributed to improving the survival duration in various types of cancer, including lung cancer [1], malignant melanoma [2,3], urinary tract cancer [4], digestive tract cancer [5], and microsatellite instability-high (MSI-H) solid tumor [6]. On the other hand, it has been reported that ICIs can damage various organs in what are known as immune-related adverse events (irAEs).

Colitis is one of the most common irAEs [7]. The incidence of irAE colitis is reported to be 0.7–1.6% with anti-PD-1 therapy, 5.7–9.1% with anti-CTLA-4 therapy, and nearly 13.6% with the combination of anti-PD-1/PD-L1 and anti-CTLA-4 therapies [8,9,10]. Thus, the likelihood of irAE colitis increases in combination therapy [11,12]. Coexisting inflammatory bowel disease (IBD) contributes to the development of severe irAE colitis [13,14].

An association between irAE colitis and prognosis in several cancers has been reported [15,16,17,18,19,20,21]. Masuda et al. [19] reported significantly longer overall survival (OS) and progression-free survival (PFS) in patients with gastric cancer receiving nivolumab with the onset of irAE colitis than in those without it. Yamada et al. [20] reported that patients with irAE colitis and gastritis achieve better OS by continuing ICI treatment for malignant melanoma. Zou et al. [21] reported that patients with irAE colitis who survived more than three months presented a better cancer response and OS. However, irAE colitis might significantly reduce the patient’s quality of life and lead to the interruption or discontinuation of the treatment.

The first-line therapy for irAE colitis is corticosteroids (CSs). In clinical settings, biologics such as infliximab (IFX) and vedolizumab (VED) are often used as an additional treatment for irAE colitis refractory to CS. However, the mechanisms and effectiveness of these biologics on irAE colitis have not been fully discussed, and there is no consensus as to the cases for which biologics should be administered. The aim of this review is to clarify the mechanism of action, effectiveness, and safety of the drugs available for irAE colitis. Mechanism-based insights may lead to appropriate treatment choices for patients with refractory irAE colitis.

We performed a literature search for original articles in the electronic database PubMed through March 2022. The key search terms were “irAE colitis” and “ICI-induced colitis.” We considered all articles that included data on the action and effect of ICI on tumors, the mechanisms of irAE, and clinical research related to ICI eligible for the review. Non-English language articles were excluded. The titles and abstracts obtained from electronic searches were scrutinized, and the full manuscripts and their citation lists were analyzed.

## 2. Pathophysiology of ICI-Induced Colitis

PD-1 is a type I membrane protein that was identified in 1992 as a cell-death-related molecule expressed on the cellular surface as a monomer [22]. PD-1 is widely expressed on immune cells, including activated T cells, B cells, monocytes, natural killer cells, and dendritic cells. Two ligands for PD-1 have been reported: PD-1 ligand-1, B7-H1 (PD-L1) [23] and PD-1 ligand-2, B7-DC (PD-L2) [24,25].

PD-L1 and PD-L2 are expressed in tumor cells and stromata, and PD-L2 contributes to suppressing T cell activity via PD-1 [26]. PD-L1 is frequently expressed in lymphocytes, the vascular endothelium, reticular fibroblasts, mesenchymal stem cells, islet cells, astrocytes, neurons, and keratinocytes [27,28]. Cytokines such as IFN-γ, TNF-α, IL-6, IL-17, IL-10, and IL-4 increase PD-L1 expression [29,30,31,32,33,34]. The binding of PD-1 to a ligand inhibits the activation of immune cells and suppresses cytokine secretion from immune cells via the activation of intracellular signaling pathways [35]. PD-L1 is overexpressed in tissues and organs affected by irAE [36]. For instance, PD-L1 expression is increased in the intestinal mucosa of patients suffering from irAE colitis.

Additionally, CTLA-4 is expressed on CD4+/CD8+ T cells, B cell subsets, and thymocytes, setting a threshold for T cell activation and preventing autoimmunity and immune hyperactivity [37,38]. Anti-CTLA-4 therapy exerts an anti-tumor effect by enhancing the T cell response and producing cytokines such as IFN-γ, an essential cytokine for the host immune response [39]. Tumors with deletions in the IFN-γ pathway gene do not benefit from anti-CTLA-4 therapy [40]. Additionally, nearly half of all patients treated with the combination of anti-CTLA-4 and anti-PD-L1 antibodies develop colitis [41], and anti-CTLA-4 therapy at a high dosage causes colitis more frequently than low-dose therapy. These clinical data suggest that CTLA-4 plays a pivotal role in maintaining intestinal homeostasis.

Sury et al. proposed four hypotheses concerning the mechanisms of irAE: (1) ICI induces T cell infiltration and complement-mediated tissue damage by directly binding to cell surface proteins such as CTLA-4 expressed in normal tissues; (2) ICI promotes the recognition or binding of T cells to either the same tumor antigen or mologous tissue antigens expressed in untargeted organ tissues; (3) ICI increases cytokine levels within affected tissues, promoting the infiltration of inflammatory molecules into off-target tissues; and (4) ICI increases the level of autoantibodies against target organs or promotes the formation of de novo autoantibodies [42,43]. There are several reports on the pathogenesis of irAE colitis. Barnes et al. [44] showed that CTLA-4 is a key factor that regulates the composition of the Foxp3 T cell population in the intestine. Luoma et al. [45] showed that the overactivation of tissue-resident CD8+ T cells plays an important role in colitis. The activation of such T cells induces the subsequent recruitment of additional CD8+ and CD4+ T cell populations from the blood. irAE colitis occurs relatively early after the administration of ICI because a large number of such tissue-resident CD8+ T cells are already present in the healthy colon. The authors also demonstrated the release of cytokines (e.g., IL-1β, IFN-γ, and TNF-α) from cytotoxic T cells (CTLs) and the high expression of chemokine receptor genes on colitis-associated T cells in patients with irAE colitis, which may be involved in the development of irAE colitis.

Various important findings have been published regarding bacteria-related mechanisms in irAE colitis. Abu-Sbeih et al. [46] reported the effect of antibiotic treatment on human irAE colitis. They showed that the use of antibiotics strongly correlated with a lower occurrence of irAE colitis while causing a more severe form of the disease. In addition, anaerobic antibiotics were more clinically harmful than aerobic antibiotics. Mouse models have also shown that anaerobic bacterial strains are involved in irAE colitis resolution [47,48,49]. However, further research on the associations between irAE colitis and the microbiome is needed.

## 3. Clinical Characteristics of ICI-Induced Colitis

The severity of irAE colitis is assessed using the Common Terminology Criteria for Adverse Events (CTCAE) [50]. Grade 1 irAE is characterized by asymptomatic or mild diarrhea. Grade 2 colitis presents as mild abdominal pain, watery diarrhea, and hematochezia. Grade 3 and grade 4 colitis entail severe abdominal pain and frequent diarrhea, which can cause intestinal obstruction, peritonitis, and intestinal perforation [51,52,53,54]. The endoscopic findings for irAE colitis are edema, a loss of vascularity, erythema, mucosal granularity and friability, erosions, and ulcers. Studies have shown no association between diarrheal grade or severity of abdominal pain and endoscopic appearance. Histopathologically, neutrophil infiltration, cryptitis, and crypt abscess are frequently observed in both UC and irAE colitis. Apoptosis has been frequently observed in irAE colitis [8,55,56,57]. These similarities suggest that the treatments proposed for UC could also be effective for irAE colitis.

According to the American Society of Clinical Oncology (ASCO) clinical practice guidelines and the National Comprehensive Cancer Network (NCCN) guidelines [58,59], grade 1 colitis can be managed without discontinuing ICI treatment by using antidiarrheal drugs. In grade 2 colitis, ICI should be discontinued until improvement to grade 1 is achieved, and systemic CSs (0.5–1 mg/kg/day) are recommended as a treatment regimen. Grade 3 or grade 4 colitis requires hospitalization and careful systemic therapy with high doses of CSs (Table 1). CSs are the first-line treatment for patients with moderate to severe irAE colitis; however, CS-refractory or -resistant cases sometimes occur. Several retrospective studies have reported that 29.3–56.3% of patients with symptomatic irAE colitis of a severity greater than grade 2 did not respond to CS treatment [51,52,60].

CS-resistant irAE colitis can lead to serious conditions such as peritonitis and intestinal perforation, which can be fatal if treatment is unsuccessful [61,62]; it is therefore urgent to establish additional treatment for CS-resistant irAE colitis. We propose treatments for irAE colitis based on the morphological and immunological similarities between irAE colitis and ulcerative colitis (UC) in clinical practice.

## 4. Treatment of irAE Colitis

### 4.1. Corticosteroids

The first-line treatment for irAE colitis, as for most other irAEs, is CSs [59]. CSs inhibit the innate and adaptive immune systems by inducing apoptosis in activated T cells and inhibiting dendritic cell maturation [63,64]. In addition, CSs inhibit the production of pro-inflammatory cytokines from activated T cells, such as IL-2 and IFN-γ [65]. Furthermore, it has been demonstrated in mouse models that CSs enhance the surface expression of PD-1 in both CD4+ and CD8+ T cells and suppress their functions [66]. These findings can explain the effectiveness of systemic CSs for irAE colitis [67].

In general, CS treatment for irAEs is temporary, and CSs should be tapered off over 4 to 6 weeks when symptoms improve [59]. However, CSs do not necessarily lead to an immediate improvement in symptoms, which might also recur during tapering. Colon ulcers, entire colon inflammation, and a high Mayo score have been reported as predictors of patients with CS-refractory irAE colitis [29,67]. In addition, the molecular characteristics of aggressive irAE colitis are increased in the presence of group 3 innate lymphoid cells (ILC3s) in the mucosa and the intense infiltration of CD4+ and CD8+ T cells [45,47,48,49,50,51,52,53,54,55,56,57,58,59,60,61,62,63,64,65,66,67,68,69]. There are also reports suggesting that specific human leukocyte antigen (HLA) expression (HLA-B*35, DRB1*11) correlates with the risk of developing irAEs [70,71]. Furthermore, Coutzac et al. [68] showed a negative correlation between mucosal TNF-α expression levels and susceptibility to CS. Sakurai et al. [72] reported that the expression of genes involved in IFN-γ signaling are increased in the intestinal mucosa of patients with CS-resistant irAE colitis.

It should be noted that the long-term, high-dose application of CSs increases the risk of complications such as osteoporosis, infections, and impaired glucose tolerance [73,74]. In addition, although CSs are effective for irAEs, there is concern that their anti-tumor effect may be decreased by adverse mechanisms such as cytokine inhibition. Several reports have indicated that the use of CSs does not affect patient survival [75,76,77]. Skribek et al. [77] examined the effect of CSs on survival outcomes in patients with non-small-cell lung cancer. In their cohort, the CS group (defined as CS ≥10 mg, ≥10 days) included 31 patients with irAE, ten of whom had irAE colitis of severity grade 2 or higher. They showed that CS administration for alleviating cancer-related symptoms was the only independent predictor of diminished survival and that CS treatment for irAE had no effect on survival. Table 2 summarizes the papers reporting an association between the use of CSs for irAE and cancer prognosis. Additionally, Faje et al. [78] reported that the survival rate decreased in patients with malignant melanoma who received high-dose CS treatment (defined as CS ≥7.5 mg, ≥2 months) for irAE hypophysitis. Therefore, whether CSs affect the survival of patients with irAE is still controversial. It is necessary to minimize CS exposure by taking into account the various complication risks and the unpredictable prognosis effects. Alternative treatment courses should be explored for irAE colitis in CS-resistant cases.

### 4.2. Infliximab

The ASCO guidelines, NCCN guidelines, and the Society for Immunotherapy of Cancer (SITC) Toxicity Management Working Group recommend IFX for CS-resistant cases [58,59,79]. IFX is an anti-TNF-α monoclonal antibody that has been reported to be highly effective in IBD (i.e., Crohn’s disease and UC). Several case reports and retrospective studies have also shown its effectiveness in irAE colitis [56,60,61,67,80,81,82,83,84]. TNF-α signaling is highly involved in cellular functions, such as cell migration, proliferation, and apoptosis.

Table 3 summarizes the previous reports on the use of IFX for treating CS-resistant irAE colitis [56,60,81,83,84,85,86,87,88,89,90]. For CS-resistance irAE colitis, IFX 5 mg/kg/dose is administered intravenously according to the usual method of administration for IBD. The duration of treatment with IFX is not clearly defined. Treatment response to IFX for irAE colitis generally occurs within a few days and symptoms resolve with one dose [56,87,87,88]. However, some patients with irAE colitis require a second dose of IFX two weeks later. There is evidence to support up to three doses (weeks 0, 2, and 6) to reduce the risk of recurrence and increase the likelihood of endoscopic/histological remission [91]. In retrospective cohort studies conducted by Hillock et al. [84] and Alexander et al. [81], the remission rate in IFX-treated CS-refractory irAE colitis was reported to be 54% and 71.4%, respectively. Alexander et al. also showed that tumor growth was more suppressed in IFX-refractory cases than in IFX-responder cases, and rectal bleeding and crypt abscesses were IFX-resistant factors. There were six case reports, with one case each of cytomegalovirus colitis, severe liver injury (requiring steroid pulse therapy), and colon perforation as adverse events [85,86,87].

Johnson et al. [92] compared the clinical observations in patients with irAE colitis who were treated with CSs alone and those who received IFX after starting CS therapy. The time until symptom resolution was remarkably shorter in patients who received IFX after CS therapy, suggesting that the early introduction of IFX should be considered. In addition, Abu-Sbeih et al. [91] and Merrill et al. [93] showed that the early introduction of IFX contributed to a shorter duration of hospitalization.

The influence of IFX on the anti-tumor effect of ICIs is controversial. Badran et al. showed that five patients with CS-resistant irAE colitis could achieve both disease control and colitis control with the combination of IFX and ICIs [94]. Lesage et al. [82] and Wang et al. [15] also reported that the use of IFX for irAE colitis did not affect survival. In contrast, Verheijden et al. [95] compared survival rates between the CS-only and the IFX-treated groups in all irAE-affected patients studied and showed that the overall survival rate decreased in the IFX-treated group. Chen et al. [96] reported that TNF-α inhibitors enhanced ICI’s anti-tumor activity by promoting cytotoxic T cell (CTL) activity and may exert a direct cancer inhibitory effect by suppressing the function of regulatory T cells (Treg). However, while there are direct effects of TNF-α inhibition on tumor formation, the long-term use of TNF-α inhibitors may block the differentiation of naïve CD8+ T cells into CTL and deplete anti-tumor CTL cells. Although there have been no reports that IFX administration for irAE colitis directly exacerbates the primary disease, the long-term use of IFX should be avoided, and IFX administration should be discontinued when remission is achieved.

### 4.3. Vedolizumab

VED is an IgG1 monoclonal antibody that specifically binds to α4β7 integrin on activated T cells. It inhibits the entry of activated T cells into intestinal tissue by blocking the interaction with mucosal addressin cell adhesion molecule-1 (MAdCAM-1), which is selectively expressed in intestinal vascular endothelial cells [97,98]. The efficacy of VED has been demonstrated in IBD [99]. Although there are fewer datasets available for VED as compared to IFX, it is presented as a treatment option next to IFX in the ASCO and NCCN guidelines [58,59].

Table 4 summarizes previous reports of VED usage in CS-refractory irAE colitis [71,73,99,100,101,102,103,104]. In each report, VED (300 mg/day) was administered intravenously at weeks 0, 2, and 6 and every 8 weeks thereafter until symptoms improved. In the cases reported by d’Apolito et al. [71] and Hsieh et al. [99], VED was administered as an alternative to IFX because of the risk of sepsis due to coexisting bone marrow suppression and infectious diseases. Abu-Sbeih et al. [103] conducted a retrospective study to examine the therapeutic effects of VED on irAE colitis refractory to CSs and/or IFX. The remission rate was 67% in patients who received IFX before VED and 95% in patients who did not receive IFX. No adverse events were observed. Despite the lack of an exact determination of the VED treatment duration, Abu-Sbeih et al. [94] reported that up to three doses (at weeks 0, 2, and 6) reduced the risk of relapse and increased the likelihood of endoscopic/histologic remission.

There have been no reports regarding the direct comparison of clinical trials between IFX and VED treatment for CS-refractory irAE colitis.

### 4.4. Other Therapeutic Agents

The therapeutic effects of mycophenolate mofetil (MMF) [30,105], calcineurin inhibitors (tacrolimus [106,107] and cyclosporine [108]), and tocilizumab [109] have also been reported.

MMF inhibits inosine-5′-monophosphate dehydrogenase (IMPDH) and exerts immunosuppressive effects by inhibiting T-cell and B-cell replication. Mir et al. [105] reported 11 cases of irAE colitis treated with MMF in combination with CSs. Out of eleven, seven patients did not develop subsequent colitis flares during CS tapering. The remaining four patients, who had relapsed, responded strongly to IFX.

Calcineurin inhibitors (CNIs) (tacrolimus and cyclosporine) bind to calcineurin by forming an intracellular complex with FK506-binding protein 12, suppressing the release of cytokines such as IL-2, TNF-α, and IFN-γ, and exhibit a robust immunosuppressive effect by inhibiting T cell activation. Calcineurin inhibitors are commonly used for patients with moderate to severe UC [110]. The British Society of Gastroenterology (BSG) and the European Society for Medical Oncology (ESMO) have recommended using tacrolimus for irAE colitis [111]. Kunogi et al. [106] reported a case in which diarrhea improved after tacrolimus administration for irAE colitis refractory to CSs, IFX, and VED. In their reports, tacrolimus was effective for irAE, but hepatic metastasis appeared three months after tacrolimus administration.

Tocilizumab, an anti-IL-6 receptor antibody, is an established treatment for moderate to severe rheumatoid arthritis (RA). IL-6 promotes inflammation via the trans-signaling pathway [112] and is known to promote tumor progression and metastasis through various mechanisms such as the activation of tumor formation pathways and the inhibition of dendritic cell differentiation [113]. Therefore, IL-6 inhibition may achieve both tumor suppression and cancer-related symptom management. Stroud et al. reported 34 cases of CS-refractory irAEs in patients who were treated with tocilizumab. Of these, only one patient had irAE colitis, and tocilizumab alleviated the symptoms without affecting survival. When using tocilizumab, it should be noted that an increased risk of intestinal perforation has been reported in clinical trials in patients with RA. In particular, patients with ulcerative lesions in the stomach or intestine who continued long-term CS treatment had an increased risk of intestinal perforation [114]. Therefore, in patients with a history of long-term CS-administration or severe gastrointestinal ulcers with irAE colitis, tocilizumab should be administered carefully.

5-aminosalicylic acid (5-ASA) is a drug commonly used for IBD that acts locally on the colonic epithelium. The anti-inflammatory effects of 5-ASA are primarily exerted by inhibiting cyclooxygenase and lipoxygenase, followed by a decrease in the production of prostaglandins and leukotrienes [115]. The nuclear receptor peroxisome proliferator-activated receptor ligand-γ (PPAR-γ), a transcription factor that inhibits TNF-α production, is activated by 5-ASA [116]. There have been reports regarding the administration of 5-ASA to patients with preexisting UC who develop irAE [117], but its efficacy is unclear.

**Figure 1:**
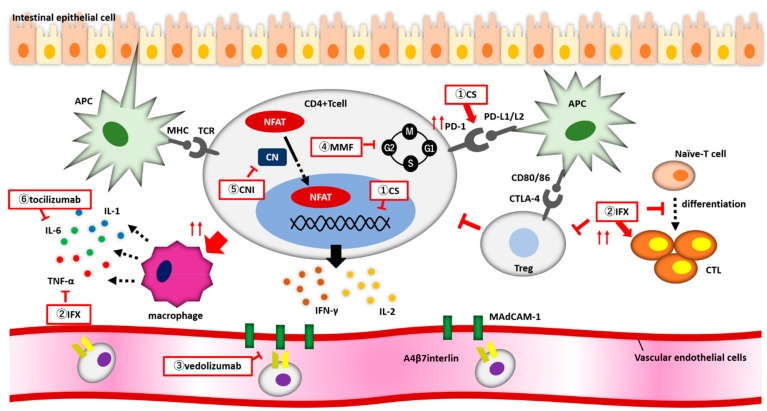
Action mechanisms of CSs and biologics for irAE colitis. (**1**) Corticosteroids (CSs) enhance PD-1 expression on the surface of CD4+ T cells, bind to the nuclear receptor of CD4+ T cells, and suppress the release of inflammatory cytokines. (**2**) Infliximab enhances CTL activity, suppresses Treg function, and inhibits naïve T cells from differentiating into CTLs. (**3**) Vedolizumab inhibits the binding of α4β7 integrin to MadCAM-1 and blocks CD4+ T cells from migrating from blood vessels into the intestine. (**4**) MMF reversibly and specifically inhibits IMPDH, and lymphocytes arrest proliferation during the G1 to S phases of the cell cycle. (**5**) Calcineurin inhibitors block NFAT from migrating into the nucleus and reduce the expression of inflammatory cytokine genes.

The action mechanisms of CSs and biologics for irAE colitis are shown in Figure 1.

## 5. Discussions

This review summarizes the available pharmacological treatments for irAE colitis, including their mechanisms and concerns.

ICI administration cannot be continued in many cases, depending on the extent of the irAE. Several guidelines recommend careful follow-up and the initiation of CS therapy after the onset of irAE colitis. However, CSs are ineffective in approximately half of patients, and there are concerns about complications associated with long-term or high-dose administration. Therefore, to minimize the risk of complications from CS exposure, it is necessary to assess the efficacy of CSs and consider alternative treatments for CS-resistant irAE colitis. However, secondary treatment for CS-refractory or CS-resistant cases has not yet been established.

The most frequently used biologic for irAE colitis is IFX, and several guidelines recommend its use for CS-resistant irAE colitis. VED, MMF, CNI, and tocilizumab have also been helpful in some cases of CS-resistant irAE colitis. Although these biologics have been reported to contribute to better outcomes in previous retrospective case studies, no extensive prospective studies have been conducted. In addition, the doses and dosing intervals are similar to those used for IBD. However, it is questionable whether these regimens are optimal for irAE colitis.

We believe that clarifying the appropriate treatment for refractory irAE colitis would simplify the management of patients receiving ICI and improve their prognosis. To date, few reports have summarized the molecular mechanisms and therapeutic outcomes of available drug therapies for irAE colitis. Therefore, prospective clinical trials that directly compare IFX with VED or other biologics would be helpful.

A limitation of this review is that it focuses only on pharmacotherapy for ICI-induced colitis. The scope of the study may be limited because we did not include topical therapy, fecal microbiota transplantation, and surgical interventions in this review.

## 6. Conclusions

The application of ICIs has revolutionized oncological treatment and improved the prognosis for many patients. However, the administration of ICIs may result in irAEs such as irAE colitis, which reduces the quality of life and leads to the discontinuation of ICI treatment despite its favorable disease prognosis. Similar to the treatment for IBD, biologics have been suggested for use in irAE colitis. Despite increasing data indicating a favorable response for irAE colitis, the efficacy and safety of biologics for irAE colitis treatment are still controversial. Further studies are required to select appropriate therapeutic agents for irAE colitis.

## Figures and Tables

**Table 1:** Grading the severity of irAE colitis based on Common Terminology Criteria for Adverse Events.

Colitis Grade	Symptoms	Management
Grade 1	Asymptomatic (clinical or laboratory findings only)	Consider applying closely monitored immunotherapy with loperamide or diphenoxylate/atropine
Grade 2	Abdominal pain, mucus, blood in stool	Consider systemic corticosteroids (1–2 mg/kg/day); if no response in 2–3 days, continue corticosteroids and consider adding infliximab or vedolizumab within two weeks
Grade 3	Severe abdominal pain, peritoneal signs	Consider inpatient supportive care; intravenous corticosteroids (1–2 mg/kg/day); if no response in 2 days, continue corticosteroids and strongly consider adding infliximab or vedolizumab within two weeks
Grade 4	Severe and persistent abdominal pain, fever, ileus, life-threatening complications such as perforation and peritonitis	
Grade 5	Death	

**Table 2:** The association between CS use for irAEs and cancer prognosis.

Authors	Year	Original Disease	No. of Cases (CS-Naïve: Needed CS)	Impact of CS on Response Rate or Survival
Horvat et al. [75]	2015	melanoma	195:103	Systemic CS was not associated with OS or TTF
Weber et al. [76]	2017	melanoma	462:114	ORR was 31.8% in CS-naïve group and 29.8% in CS-needed group (*p =* 0.736); median duration of response was 22.0 months in CS-naïve group and not reached in CS-needed group
Skribek et al. [77]	2020	lung cancer	104:31	OS was 14.43 months in CS-naïve group and not reached in CS-needed group (*p =* 0.38)

CS, corticosteroid; OS, overall survival; TTF, time to treatment failure; ORR, overall response rate.

**Table 3:** Summary of the cases of infliximab use for irAE colitis.

Author	Year	No. of Cases	Age [Range]	Gender (Male: Female)	Original Disease	Therapeutic Drugs	CS Treatment Period	Number of IFX Doses until Remission Number of Doses [Range]	Patients Achieving Remission	Adverse Events
Lankes et al. [85]	2016	1	32	1:0	melanoma	combination	30 w	4	0% (0/1)	CMV colitis
Yanai et al. [56]	2017	1	51	1:0	melanoma	PD-1/L1	17 d	1	100% (1/1)	Without
Zhang et al. [86]	2019	1	79	1:0	prostate cancer	combination	25 d	1	100% (1/1)	Liver disorders
Callens et al. [87]	2019	1	63	0:1	lung cancer	PD-1/L1	3	1	0% (0/1)	Perforation of the large intestine
Miyahara et al. [83]	2020	1	72	1:0	melanoma	combination	ND	1	100% (1/1)	Without
Paparoupa et al. [88]	2020	1	54	0:1	melanoma	combination	2 m	17	100% (1/1)	Without
Minor et al. [89]	2009	3	57 [47,48,49,50,51,52,53,54,55,56,57,58]	3:0	melanoma	CTLA-4	ND	2 [1,2]	100% (3/3)	Without
O’Connor et al. [90]	2016	4	ND	ND	melanoma	ND	ND	ND	100% (4/4)	Without
Jain et al. [60]	2017	9	ND	ND	melanoma	CTLA-4	ND	1 [1,2]	100% (9/9)	Without
Hillock et al. [84]	2017	13	64 [40,41,42,43,44,45,46,47,48,49,50,51,52,53,54,55,56,57,58,59,60,61,62,63,64,65,66,67,68,69,70,71,72,73,74,75,76,77,78,79,80,81,82,83,84,85,86]	6:7	melanoma: 13	CTLA-4	ND	1 [1,2,3]	54% (7/13)	Without
Alexander et al. [81]	2021	127	59 [26,27,28,29,30,31,32,33,34,35,36,37,38,39,40,41,42,43,44,45,46,47,48,49,50,51,52,53,54,55,56,57,58,59,60,61,62,63,64,65,66,67,68,69,70,71,72,73,74,75,76,77,78,79,80,81,82,83,84,85,86,87,88]	73:54	melanoma: 90, kidney cancer: 15, lung cancer: 7, urinary tract cancer: 8, others: 7	PD-1/L1:40, CTLA-4:21 combination: 66	ND	ND	71.4% (75/105)	Without

ND: not described.

**Table 4:** Summary of the cases of vedolizumab use for irAE colitis.

Author	Year	No. of Cases	Age [Range]	Gender (Male: Female)	Original Disease	Therapeutic Drugs	Duration of CS Treatment	Administration of IFX	Number of Vedolizumab Doses until Remission [Range]	Patients Achieving Remission
Hsieh et al. [99]	2016	1	69	1:0	melanoma	CTLA-4	33 w	no	3	100%
Diana et al. [100]	2018	1	62	0:1	melanoma	combination	ND	yes	2	100%
Randhawa et al. [101]	2019	1	27	0:1	melanoma	combination	55 d	yes	3	100%
Stone et al. [73]	2021	1	68	0:1	lung cancer	PD-L1	6 w	no	ND	100%
d’Apolito et al. [71]	2022	1	44	0:1	melanoma	PD-1/L1	15 d	no	3	100%
Bergqvist et al. [102]	2017	7	55 [40,41,42,43,44,45,46,47,48,49,50,51,52,53,54,55,56,57,58,59,60,61,62,63,64,65,66,67,68,69,70,71]	4:3	melanoma: 6, lung cancer: 1	CTLA-4: 6, PD-1/L1: 1	57 d (52–92)	no: 6, yes: 1	2 [2,3,4]	100%
Abu-S et al. [103]	2018	28	63	20:8	melanoma: 7, urinary tract cancer: 7, prostate cancer: 4, others: 10	CTLA-4: 8, PD-1/L1: 12, combination:8	96 d	yes: 9, no: 19	3 [1,2,3,4]	86%
Zou et al. [104]	2021	62	63 [49,50,51,52,53,54,55,56,57,58,59,60,61,62,63,64,65,66,67,68,69,70,71]	41:21	melanoma: 10, urinary tract cancer: 23, lung cancer: 10, others: 19	CTLA-4: 6, PD-1/L1: 38, combination:18	35 d (27–43)	no	3	89%

ND: not described.

## Data Availability

Not applicable.

## Abbreviations

PD-1, programmed cell death 1; PD-L1, programmed cell death ligand 1; CTLA-4, cytotoxic T-lymphocyte antigen 4; APC, antigen-presenting cell; Treg, regulatory T cell; CTL, cytotoxic T cell; MHC, major histocompatibility complex; TCR, T cell receptor; CS, corticosteroid; IFX, infliximab; MMF, mycophenolate mofetil; IMPDH, inosine-5′-monophosphate dehydrogenase; CNI, calcineurin inhibitor; MAdCAM-1, mucosal addressin cell adhesion molecule-1; NFAT, nuclear factor of activated T cells; IFN-γ, interferon-γ; IL-1, interleukin-1; IL-2, interleukin-2; IL-6, interleukin-6; TNF-α, tumor necrosis factor-α.
